# Mitral valve replacement complicated by iatrogenic left ventricular outflow obstruction and paravalvular leak: case report and review of literature

**DOI:** 10.1186/s12872-015-0108-z

**Published:** 2015-10-09

**Authors:** Justin Z. Lee, Kai R. Tey, Ahmad Mizyed, Charles T. Hennemeyer, Rajesh Janardhanan, Kapildeo Lotun

**Affiliations:** Department of Internal Medicine, University of Arizona, 1501 N. Campbell Avenue, RM 6336, Tucson, AZ 85724-5040 USA; Department of Cardiovascular Diseases, University of Arizona, 1501 N. Campbell Avenue, RM 6336, Tucson, AZ 85724-5040 USA; Department of Radiology, University of Arizona, 1501 N. Campbell Avenue, RM 6336, Tucson, AZ 85724-5040 USA

**Keywords:** Left ventricular outflow tract obstruction, Paravalvular leak, Septal ablation

## Abstract

**Background:**

Left ventricular outflow tract (LVOT) obstruction and paravalvular leak (PVL) are relatively uncommon, but are serious complications of prosthetic valve replacement.

**Case presentation:**

We present a case that displays the unique therapeutic challenges of treating a patient who developed both LVOT obstruction and mitral PVL after undergoing surgical aortic and mitral valve replacement (MVR). We also describe the use of alcohol septal ablation and albumin-glutaraldehyde (BioGlue) for septal ablation to percutaneously treat the patient’s LVOT obstruction, followed by use of an Amplatzer vascular plug for percutaneous closure of an antero-medial mitral PVL associated with severe regurgitation.

**Conclusion:**

Percutaneous interventional management of these entities may be considered as an initial therapeutic option, especially in high-risk patients with significant morbidity and mortality of repeat surgical operations.

## Background

The outcome of patients with valvular heart disease has significantly improved since valve replacement surgery was introduced in the 1960’s. Despite technological advancements in valve function and design, prosthetic valve replacement is associated with significant adverse events, including valve thrombosis, thromboembolism, bleeding, and endocarditis [1]. Paravalvular leak (PVL) is also a well-recognized complication with a reported incidence of 5–15 % in the United States [2]. However, this may have been underestimation due to the lack of systematic screening as well as difficulty in diagnosis due to obscured view on color flow Doppler images secondary to annular calcifications [2]. Left ventricular outflow tract (LVOT) obstruction is a relatively rare but serious complication of prosthetic valve replacement, with reported incidence of 4–5 % post mitral valve repair [3]. We describe a patient who presented with both PVL and LVOT obstruction after surgical aortic and mitral valve replacement (MVR), and share our experience of the management of this case with percutaneous techniques under guidance of advanced imaging modalities.

## Case presentation

**Fig. 1:**
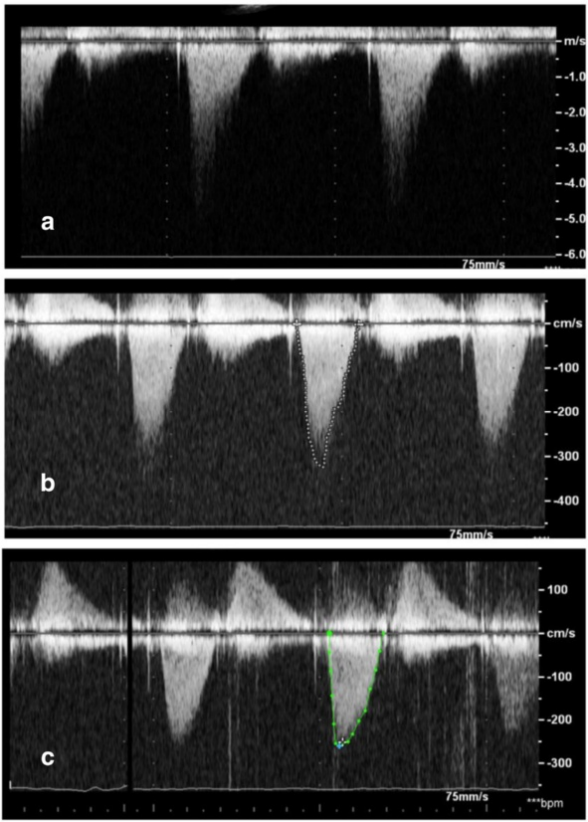
Intra-procedural Trans-esophageal Echocardiography. **a** Pre-procedure continuous wave Doppler velocity tracing showing elevated gradient across LVOT which is characteristic of dynamic outflow obstruction. **b** After alcohol septal ablation, continuous wave Doppler velocity still showed elevation of gradient across LVOT. **c** Continuous wave Doppler velocity showing reduction in gradient across LVOT after injection of glue

**Fig. 2:**
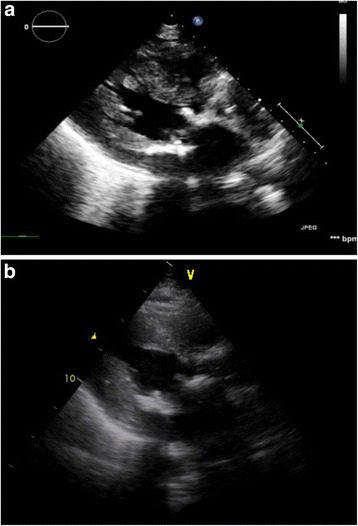
2-D transthoracic echocardiography. **a** Pre-septal ablation. **b** Post-septal ablation showing septal scar and remodeling with relief of LVOT obstruction

**Fig. 3:**
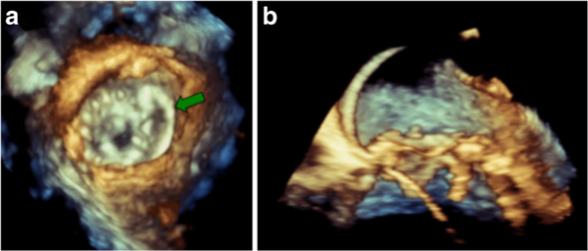
Real-time 3-D Trans-esophageal echocardiography. 3-D Zoom view on 3-D TEE pre-deployment showing (**a**) antero-medial paravalvular defect in the mitral valve (*green arrow*) and (**b**) wire passing through paravalvular leak

A 67-year-old lady with hypertension and type-2 diabetes mellitus was diagnosed with severe aortic and mitral stenosis after a syncopal event. She subsequently underwent surgical bioprosthetic aortic valve replacement at an outside community hospital, and the reason for a single valve surgery was unclear. Post-operatively, transthoracic echocardiography (TTE) showed an elevated trans-valvular gradient across the bioprosthetic aortic valve. She was then referred for surgical re-intervention of the aortic bioprosthetic valve as well as mitral valve replacement. A few days after being discharged from the hospital, she developed progressive dyspnea with New York Heart Association (NYHA) Class IV symptoms along with hemolytic anemia requiring multiple blood transfusions. Color flow Doppler on TTE demonstrated flow acceleration through the LVOT secondary to obstruction with increased peak gradient of 80-mmHg [Fig. 1a]. There was severe concentric left ventricular hypertrophy (basal septal thickness approximately 20-mm) [Fig. 2a]. Positioning of the bioprosthetic mitral valve in combination with severe septal hypertrophy appeared to be the etiology of the LVOT obstruction. During her evaluation, real-time 3D Trans-esophageal echocardiography (TEE) was also performed confirming the LVOT obstruction and showing a PVL located along the antero-medial aspect of the bioprosthetic mitral valve associated with severe mitral regurgitation [Fig. 3].

**Fig. 4:**
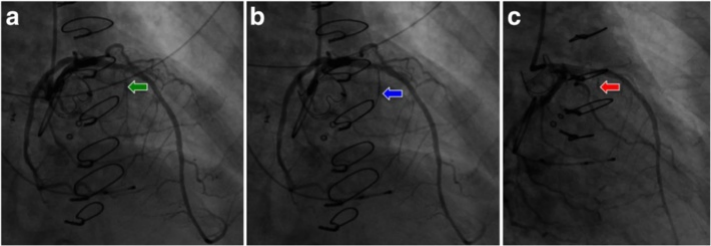
Coronary angiography. Coronary angiography performed showing (**a**) location of the septal perforator, (**b**) wire in the septal and (**c**) balloon inflation in the septal branch

Given the patient’s acutely decompensated clinical state and high operative risk for “re-do” valve surgery, the decision was made to take the patient to the cardiac catheterization laboratory for attempted percutaneous management of the LVOT obstruction with alcohol septal ablation (ASA). The procedure began with placement of a temporary pacemaker through a 5 French sheath via the right internal jugular vein approach. The left coronary artery was then engaged with an EBU 3.5 guide catheter (Medtronic, Danvers, MA) followed by passage of 1.5 mm × 6.0 mm over-the-wire (OTW) angioplasty balloon into the first septal perforator artery using an Asahi Prowater wire (Abbott, Abbott Park, IL) [Fig. 4a, b, and c]. The balloon was then inflated and selective angiography was performed through the OTW balloon to confirm that there was no extravasation of contrast into the left anterior descending artery (LAD) and evaluated the area of myocardium to be ablated by both angiography and myocardial contrast echocardiography. Following confirmation that the septal perforator supplied the septal myocardium of interest, a slow injection of 2 mL of absolute alcohol was infused over a period of 10 min. A second slow injection of 2 mL of absolute alcohol was performed due to a persistently elevated LVOT gradient. Despite two injections of absolute alcohol, intra-procedural TEE showed a residual peak gradient across the LVOT of 50-mmHg [Fig. 1b]. At this point, the decision was made to inject 1 mL of BioGlue (Cryo Life, Kennesa, GA) - purified bovine serum albumin and glutaraldehyde [Fig. 2b] which reduced the peak LVOT gradient to 25-mmHg [Fig. 1c]. After BioGlue injection, the patient developed complete heart block and was transvenously paced for the remainder of the procedure, and ultimately required placement of a dual chamber pacemaker. There was no other complication subsequent to the procedure.

**Fig. 5:**
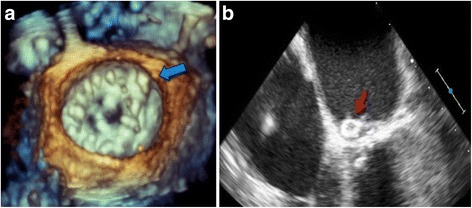
Intra-procedural Trans-esophageal echocardiography. **a** 3-D Zoom view on 3-D TEE post-deployment showing final position of Amplatzer vascular plug II (*blue arrow*). **b** 4-Chamber view on 2-D TEE showing Amplatzer vascular plug in situ (*red arrow*)

**Fig. 6:**
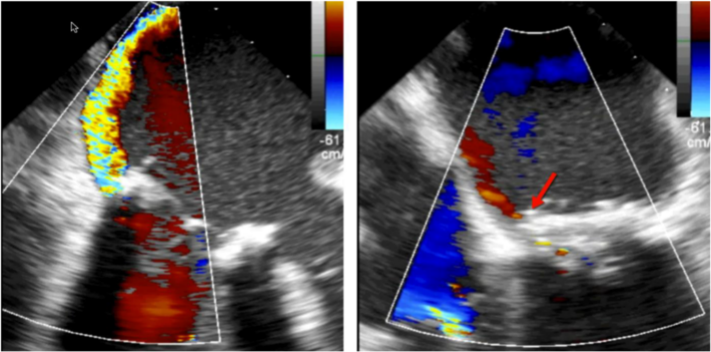
Trans-esophageal echocardiography with color Doppler. Left: Pre-procedure: Antero-medial mitral paravalvular leak associated with moderate-severe mitral regurgitation. Right: Post deployment of Amplatzer vascular plug (#red arrow) showing significant reduction of mitral regurgitation

After the LVOT obstruction was relieved, there was persistence of the PVL. Despite being symptomatically improved, she still had residual symptoms and persistent hemolytic anemia with hemoglobin in the 8 g/dl range. Thus, two weeks after the septal ablation, patient was brought back to the catheterization laboratory for repair of the PVL. Access was obtained in the right common femoral vein. An 8.5-French SRO guiding sheath (St Jude Medical, Saint Paul, MN) was advanced over the wire to the right atrium and puncture of the interatrial septum was performed under TEE guidance using a Baylis trans-septal needle (Baylis Medical, Burlington, MA). The needle was removed and a 0.032” Cook wire (Cook, Bloomington, IN) was advanced to the left atrium. The SRO sheath was then exchanged for an 8.5-French Agilis catheter (St Jude Medical, Saint Paul, MN) followed by a 6-French JR4 catheter advanced into the left atrium through the Agilis Sheath. A 0.035” Terumo floppy Glidewire (Terumo Interventional Systems, Somerset, NJ) was guided across the PVL into the left ventricle. The Agilis catheter was removed and the JR4 catheter advanced to the left ventricle (LV). An 8 mm Amplatzer Vascular Plug II (AVP II) (St Jude Medical, Saint Paul, MN) was positioned across the PVL under real-time 3D-TEE guidance and was successfully deployed [Fig. 5a and b]. Color flow Doppler on TEE confirmed reduction in mitral regurgitation from severe to mild (Fig. 6). Post procedure, patient continued to do well with no recurrent heart failure symptoms, and there was resolution of hemolytic anemia.

## Discussion

LVOT obstruction and PVL are recognized complications following valve replacement surgeries. However, MVR complicated by both LVOT obstruction and PVL has not previously been reported. Our case is unique not only because the MVR was complicated by LVOT obstruction and PVL but also because both entities were successfully treated percutaneously, avoiding the need for repeat of high-risk surgery.

Classically, LVOT obstruction occurs in the setting of hypertrophic obstructive cardiomyopathy, but it can also be associated with left ventricular hypertrophy, mitral valve abnormalities, or the use of inotropes. The narrowing of left ventricular outflow tract during systole, coupled with Venturi effect on the anterior mitral valve leaflet results in systolic anterior motion (SAM). Systolic septal bulging into LVOT causes triad of symptoms of dyspnea, angina and dizziness. The presence of resting obstruction, defined as peak LVOT gradient >30 mmHg is an important predictor for development of sudden cardiac death or progression to heart failure [4]. Documented risk factors that predispose patients to SAM include small left ventricular cavities with hyperdynamic function, narrow aorto-mitral angle, bulging left ventricular septum, and an undersized annuloplasty ring [5, 6].

LVOT obstruction can be managed medically or invasively with surgical or percutaneous methods depending on the etiology and severity. Medical management, such as beta-adrenoceptor blockade, volume loading, and phenylephrine, have been reported to be sufficient in patients with mild to moderate LVOT obstruction [7].

For severe LVOT obstruction, surgical myectomy remains the gold standard therapy [7]. Percutaneous ASA may be an alternative option to relieve symptoms due to LVOT obstruction. In this catheter-based procedure, alcohol is injected into the septal perforator branch with the aim of creating ischemia and infarction within the septal myocardium of the LVOT. ASA has been shown to effectively reduce the pressure gradient across the left ventricular outlet tract. Dabrowski et al. showed that in a group of patients with initial mean LVOT peak gradient (PG) of 123 ± 33 mm Hg, the pressure gradient across the LVOT decreased to an average of 52 ± 37 mm Hg (*p* < 0.0001) at three months after ASA and to 37 ± 28 mm Hg (*p* < 0.0001) at six months after ASA [8]. Procedural success was defined as ≥50 % reduction in the peak LVOT gradient observed at rest or a final residual resting gradient of <20-mmHg in the absence of death or need for emergency surgery [9].

A meta-analysis by Agarwal et al. that compared the outcomes of ASA and myectomy showed that ASA and myectomy had similar mortality [10]. ASA has been associated with higher post procedure complication of conduction abnormalities and higher post intervention LVOT gradient compared to myectomy. There was no significant difference in other outcomes such as post-intervention functional status, improvement in NYHA functional class, ventricular arrhythmia occurrence, re-interventions performed, and post-procedure mitral regurgitation [10].

Our patient was deemed to be too high risk for surgical re-intervention. Furthermore, the presence of an aortic prosthesis complicates surgical management of LVOT obstruction as surgical septal myectomy is traditionally performed via the trans-aortic approach. ASA was performed as described, but there was no adequate reduction in the pressure gradient across the LVOT. Thus, the decision was made to proceed with injection of BioGlue (Cryo Life, Kennesaw, GA). BioGlue consists of purified bovine serum albumin (BSA) and glutaraldehyde and is commonly used as adjunct in hemostasis in open surgical repair of large vessels. This case demonstrates the feasibility of the off-label use of BioGlue to induce septal myocardial ischemia/infarction in the treatment of LVOT obstruction due to septal hypertrophy. Our patient went into complete heart block after the injection of BioGlue requiring eventual permanent pacemaker placement. Permanent pacemaker is a known complication of ASA with an incidence of approximately 13–20 % [9, 11], but the incidence of bradyarrythmias requiring pacemaker placement after BioGlue injection in these situations is not known. Furthermore the safety of using BioGlue in these situations has not been established and further studies are necessary to evaluate its use for septal ablation in hypertrophic cardiomyopathy, particularly in the sequential use of BioGlue following alcohol-septal ablation.

Despite successful percutaneous management of our patient’s LVOT obstruction, she continued to be symptomatic from the severe mitral regurgitation associated with her PVL. Although most cases of PVLs are asymptomatic, surgical or percutaneous repair is indicated when it is associated with heart failure or persistent hemolytic anemia requiring blood transfusions. Surgical intervention is recommended in patients with symptomatic PVL, though reoperation is associated with mortality rates approaching 16 % [12]. Thus, a less invasive percutaneous approach may be an attractive alternative to open surgery. Paramitral valvular leak can be approached via antegrade transseptal, retrograde transaortic, or retrograde transapical approach. Although several studies have demonstrated the safety and efficacy of percutaneous closure of PVL [13], there are limited reports of this intervention in the presence of double prosthetic valves. In a case reported by David et al., they described a left ventricular trans-apical approach to access the PVL in a patient with double prosthetic valves [14]. In our patient, we successfully utilized an antegrade approach via a trans-septal puncture.

Even in the presence of a single prosthetic valve, percutaneous PVL repair is technically challenging and success depends on the appropriate device selection, which requires balance between completeness of the defect coverage without interfering with the valve leaflets and surrounding structures. Currently, there are no devices designed specifically to meet the unique anatomic and physiologic properties of PVL. Instead, devices that have been approved for the closure of other cardiovascular defects (e.g., patent ductus arteriosus, atrial septal defect, ventricular septal defect) by the U.S. Food and Drug Administration have been used in an off-label manner in the closure of PVL. Among noted reasons for procedural failures include prosthetic leaftlet impingement, presence of residual moderate or severe mitral regurgitation, and inability to cross defect with delivery sheath or guidewire [13]. Documented 30-day all-cause mortality was 1.7 to 5.4 % [13, 15].

Due to the complexity of the procedure, real time 3-dimensional (3-D) TEE is important to reduce procedure failures. 3-D imaging allows clear visualization of the paravalvular areas and appreciation of the spatial location of the defect in relation to the other structures. This facilitates navigation of the catheter and also deployment of the device. Besides, the use of 3-D TEE also allows us to detect procedural complications in real time, as well as to assess the immediate result of the implantation of the device.

This case demonstrates the successful use of BioGlue for septal ablation in severe LVOT obstruction and percutaneous closure of anteromedial mitral PVL via antegrade approach in the same patient in the presence of bio-prosthetic mitral and aortic valves utilizing real-time 3-D TEE guidance. Surgical repair has traditionally been considered the gold standard for management of patients with such complications. The significant morbidity and mortality associated with repeat surgery, however, is a major concern in these high-risk patients. More studies are needed to determine if BioGlue injection for septal ablation would be an effective alternative to alcohol for the treatment of LVOT obstruction due to septal hypertrophy. In terms of percutaneous PVL closure, as more experience is gained and with the development of specific closure devices for PVL, percutaneous closure will become a more standard approach.

## Conclusion

Percutaneous interventional management of LVOT obstruction and mitral PVL as a complication of surgical aortic and mitral valve replacement may be considered as an initial therapeutic option, especially in high-risk patients with significant morbidity and mortality of repeat surgical operations.

## Consent

Written informed consent was obtained from the patient for publication of this Case report and any accompanying images. A copy of the written consent is available for review by the Editor of this journal.

## Abbreviations

LVOT: Left ventricular outflow tract
PVL: Paravalvular leak
MVR: Mitral valve replacement
TTE: Transthoracic echocardiography
NYHA: New York Heart Association
TEE: Trans-esophageal echocardiography
ASA: Alcohol septal ablation
LAD: Left anterior descending artery
OTW: Over-the-wire
SAM: Systolic anterior motion
PG: Peak gradient
